# Investigation of Single-Event Upset in Graphene Nano-Ribbon FET SRAM Cell

**DOI:** 10.3390/mi14071449

**Published:** 2023-07-19

**Authors:** Naheem Olakunle Adesina

**Affiliations:** Division of Electrical and Computer Engineering, Louisiana State University, Baton Rouge, LA 70803, USA; nadesi1@lsu.edu

**Keywords:** graphene nano-ribbon FET, single-event upset, SRAM, stability, FinFET

## Abstract

In recent years, graphene has received so much attention because of its superlative properties and its potential to revolutionize electronics, especially in VLSI. This study analyzes the effect of single-event upset (SEU) in an SRAM cell, which employs a metal-oxide semiconductor type graphene nano-ribbon field effect transistor (MOS-GNRFET) and compares the results with another SRAM cell designed using a PTM 10 nm FinFET node. Our simulations show that there is a change in the data stored in the SRAM after a heavy ion strike. However, it recovers from radiation effects after 0.46 ns for GNRFET and 0.51 ns for FinFET. Since the degradation observed in Q and Qb of GNRFET SRAM are 2.7X and 2.16X as compared to PTM nano-MOSFET, we can conclude that GNRFET is less robust to single effect upset. In addition, the stability of SRAM is improved by increasing the supply voltage V_DD_.

## 1. Introduction

The reliability issue for electronic systems is gaining more attention because of the advancement and scaling down of technology nodes. Continuous device shrinking, increased integration levels, extremely low operating voltage, structural changes, high speeds, etc., have caused semiconductors or other related 2D devices to be more sensitive to radiation, consequently leading to electronic system failures and malfunction. Hence, the need to investigate radiation and its impacts on the reliability of VLSI design.

SRAM is an essential building block of a memory unit, which is usually designed for high-speed and low-power applications. It is characterized by high integration capability, fast storage speed, and compatibility with CMOS. In a radiation environment, such as space, radiation particles (e.g., alpha particles, protons, neutrons, or other heavy ions) cause a decrease in critical charge and capacitance of SRAM, thus making them more susceptible to single-event upset (SEU) [1]. An electron–hole pair is generated along the path of a charged particle in a semiconductor device, which results in a collected charge (Q_coll_). The Q_coll_ can change the state of the register, latch, a memory cell or flip flops if it exceeds the threshold value, critical charge (Q_crit_). Several pieces of research have been conducted to investigate the soft error caused by the radiation effect. Li et al. and Mahyuddin et al. introduced a strike at the circuit node with a transient current source to emulate SEU [2]. Similarly, the SEU effect in III-V Hetero-junction TFET, III-V FinFET and Si FinFET have been investigated using circuit simulation [3]. The soft error performance before and after radiation in DG TFETs 6T SRAM cells have also been studied. Xiang et al. showed the effect of single-event transient (SET) in a phase-locked loop and proposed an SET-hardened structure to improve the irradiation resistance of PLL [4,5]. Since the transistor device in this work is graphene-based, it is important to highlight the effects of radiation on graphene and graphene-based materials. Similar to Si metal-oxide-semiconductor FETs, graphene FETs are also susceptible to oxide charge trapping, which results in negative voltage shifts in the I-V curve [6]. Zhang et al. showed that the graphene layer of the GFET test structure became chemically p-type doped as a result of oxygen absorption during X-ray exposure [7]. In ref. [8], it was observed from the experiment that the defect due to radiation near the SiO_2_/graphene channel interface caused a scattering mechanism and degradation of carrier mobility. Note that it has been shown theoretically and experimentally that graphene nano-ribbon FET (GNRFET) can potentially replace planar CMOS and FinFET [9]. GNR has a finite band gap with good semiconductor properties; the GNR transistor exhibits low sub-threshold swing and high on/off ratio, which makes it suitable for logic and low power applications. A brief review and analyses of GNRFET will be discussed later in this work. 

In this paper, we present the device-level characteristics of GNRFET, study and compare SRAM circuits made of MOS-GNRFETs and FinFETs and investigate their soft error performance.

## 2. GNRFET Device Simulation and Characterization

Graphene nano-ribbon (GNR) FETs are made from GNRs, which are 1D nano-sized graphite layers or strips of graphene with superlative electronic properties. Graphene, in its pristine condition, has no bandgap and causes poor on/off current ratio and subthreshold; thus, it is not suitable for digital applications. However, its width can be patterned and modified to a few nanometers 1D GNR, thereby creating a finite gap that is required for a semiconductor. The width of GNR is inversely proportional to the induced bandgap; GNRFET switch-ability can be improved by increasing the width of the nano-ribbon. Certain variability and defects can emerge from oxide thickness, GNR width, and line of edge roughness (LER) that affect the performance of GNRFET. The different planar structures and models of MOS-GNRFET and SB-GNRFET are already presented in ref. [10], we have chosen MOS-GNRFET in this work because of its high on/off current ratio. In addition, it is more robust to process variation, operates based on thermionic emission and will compare fairly with FinFET technology, monotonic I-V curves and no voltage shifting. Table 1 summarizes the HSPICE model of n-/p-type GNRFET. It consists of a four-carbon armchair chirality graphene nano-ribbon of equal width, W_ch_, with equal spacing, 2 W_sp_. The width of a GNR is primarily defined by the number of dimer lines (N) in the lattice structure, which is given as W_ch_ = $\sqrt{3}\mathrm{dcc}\left(\frac{N+1}{2}\right)$ where the lattice constant, dcc  = 0.142 nm.

Finally, the gate width is computed as W_G_ = (2 W_sp_ + W_ch_) $\times \;n_{\mathrm{Rib}}$, where $n_{\mathrm{Rib}}$ is the number of ribbons. The range of values of these parameters is presented in Table 1. However, the minimum channel length L_ch_ = 10 nm is chosen, and the other parameters of the GNRFET model are scaled to match the PTM libraries. The standard n-MOS GNRFET was simulated using the T-SPICE EDA tool with the syntax: XDevice Drain Gate Source Sub gnrfetnmos <=15, N = 12, L_ch_ = 10 nm, T_ox_ = 0.5 nm, d_op_ = 0.001, sp =2 nm, p = 0> and this also applies to p-type. Each terminal is connected to the appropriate bias, and the outputs are probed accordingly.

**Figure 1:**
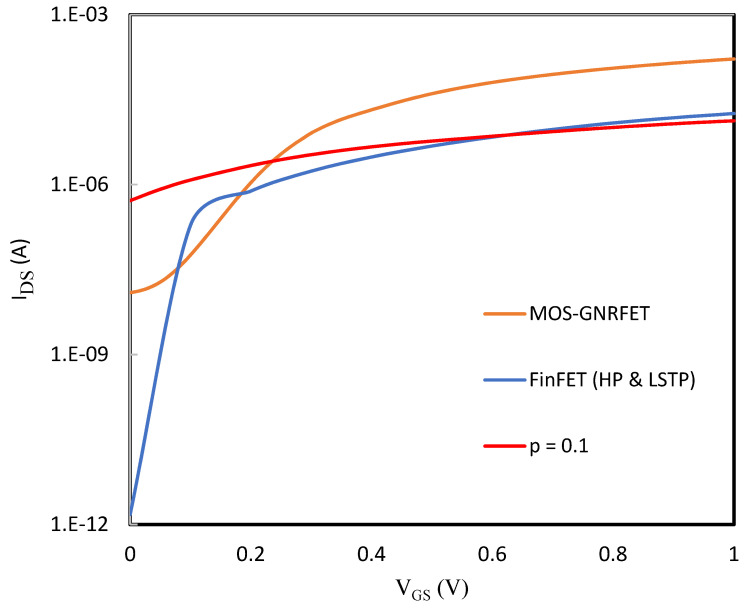
I_DS_ vs. V_GS_ for an ideal and non-ideal MOS-GNRFET, high-performance and low-stand-by power Si-CMOS.

For the transistor level characteristics, MOS-GNRFET works well with a nominal V_DD_ = 0.5 V. Figure 1 shows the curve of MOS-GNRFET and 10 nm HP and LSTP Si-CMOS n-type transistors from PTM. For a fairer comparison, we scaled the GNRFET model to match the PTM libraries. The minimum recommended nominal V_DD_ = 0.75 V is chosen for PTM devices.

From the plot, the ideal MOS-GNRFET has the highest I_on_ while PTM nano-MOSFET Si-CMOS models have better I_off_ because of multiple gates around their channels, and their respective current ratios are given in Table 2. As the edge roughness probability is increased to 10%, the I-V curve becomes worse in both I_on_ and I_off_, which indicates a degradation in the performance of the device.

**Figure 2:**
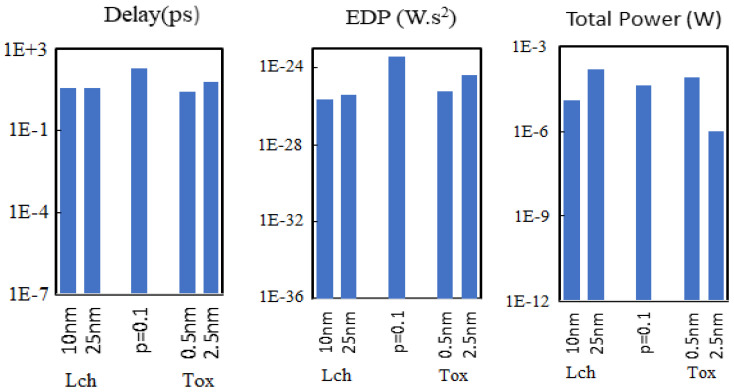
Delay, EDP, and total power vs. Lch, Tox and p. Please note that the device parameters of MOS-GNRFET are set to default values [10]. For Tox variation, Lch is set to 10 nm while Tox is set to 0.5 nm for Lch variation.

Here, we analyze the properties of an inverter designed with MOS-GNRFETs under V_DD_ = 0.5 V. Graphene nano-ribbon FET is sensitive to the major sources of variation, e.g., process, voltage, and temperature (PVT) that affect its performance. It is, however, important to check our design for different corners. During the fabrication of a transistor device, the process variation such as oxide thickness and doping concentration can vary slightly from the target specification, thereby translating to faster or slower devices. Similarly, variation in supply voltage has an effect on the current drivability, and temperature can also alter the resistance of the device. Figure 2 shows the impact of channel length, oxide thickness and line of edge roughness. Although delay increases slightly with Lch, Tox has a more significant impact on the speed of MOS-GNRFET. Similarly, a higher thick oxide reduces the gate leakage, and the total power is equally reduced.

The average delay was estimated by taking the time difference for rising and falling edges of both input and output between 10 and 90% V_DD_, and these rise and fall times are averaged to obtain the propagation delay. Similarly, the energy–delay product is calculated as EDP = (energy_1 + energy_2) × delay, where energy_1 and energy_2 are the energy consumed when the output rise and falls, respectively.

**Figure 3:**
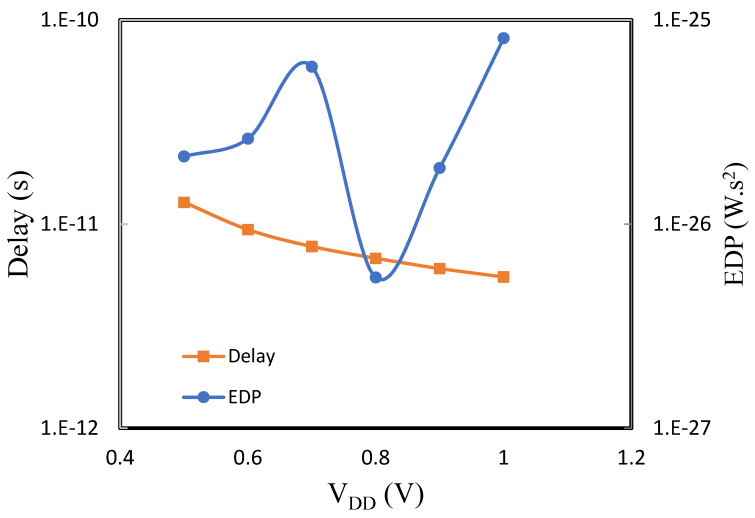
Delay and EDP vs. supply voltage for GNRFET.

**Figure 4:**
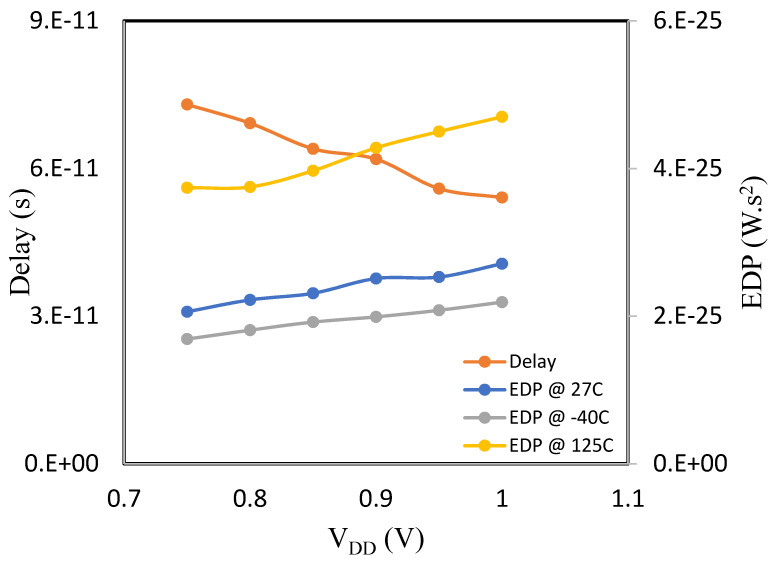
Delay and EDP vs. supply voltage for FinFET.

The delay in Figure 3 and Figure 4 decreases with V_DD_ because the drive current increases, which largely affects the rate at which the CMOS capacitive load is charged or discharged. Unlike delay, the EDP is non-monotonic, which is inconsistent with the model behavior [7]. Even though the current conduction in the MOS-GNRFET model is both thermionic and BTBT (band-to-band tunneling), its transistor properties are weakly dependent on temperature, similar to the tunnel field effect transistor.

Since the least operational V_DD_ for GNRFET is lower than FinFET, it is a bit difficult to fairly compare their performance. Nevertheless, we can deduce that GNRFET-based inverter has higher speed and consumes less power. Moreso, EDP in FinFET inverter is monotonic and increases with temperature.

## 3. Single-Event Upset in SRAM Cell

**Figure 5:**
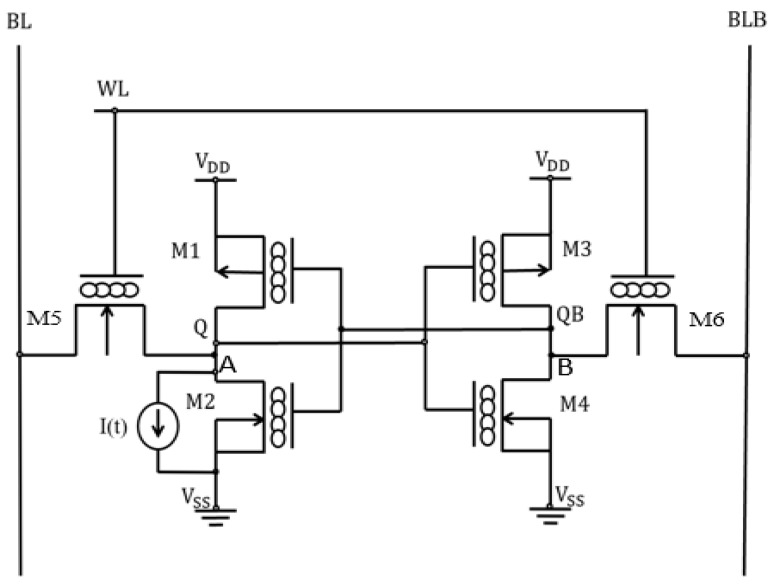
6T SRAM with single-event upset.

In this work, we employed the conventional 6T SRAM cell topology in Figure 5, which comprises the storage or memory transistors (M1–M4) and access transistors ATs (M5–M6) constructed with n- and p-type MOS-GNRFET. Transistors M5 and M6 are sized optimally to improve read and write ability and access time.

(1)    CR=(WL)1(WL)5=(WL)2(WL)6=W1W5=W2W6 

(2) PR=(WL)1(WL)5=(WL)2(WL)6=W1W5=W2W6

We adopted the strong drive transistors, medium access, and weak pull-up transistors, whereby CR (drive-to-access transistor ratio) is set equal to PR (pull-up-to-access transistor ratio). WL denotes the control input signal applied to the gates of ATs, while BL and BLB function as input or output lines depending on the mode of SRAM. Equation (3) represents the single-event upset modeled with double exponential transient time current injected at sensitive nodes A and B, and the total charge collected is given as:

(3) $$I\left(t\right)=I_{0}\left[\exp\left(\frac{-t}{\tau_{\alpha }}\right)-\exp\left(\frac{-t}{\tau_{\beta }}\right)\right]$$

(4) $$Q_{\mathrm{Coll}.}=\int_{0}^{T_{\mathrm{Coll}.}}i_{\mathrm{inj}}\left(t\right)$$

where the magnitude of the pulse current, $I_{0}$ = 5 mA, $\tau_{\alpha }$ = 20 ps and $\tau_{\beta }$ = 40 ps are time constants, $Q_{\mathrm{Coll}.}$ and $T_{\mathrm{Coll}.}$ are collected charge and collection time, respectively.

In graphene, SEU occurs when the energetic particle is incident on either of sensitive nodes A or B, and the charge builds up in the substrate. Because of reduced probabilities of interaction between graphene and ions, it creates defects such as traps, generation, and recombination sites [11]. Although the substrate defects occur mainly at the interface, there may be secondary defects inside the channel that directly influence its electronic properties. There is also a slight shift in Dirac point and a change in its field-effect mobility; therefore, the performance of GNRFET degrades with radiation.

**Figure 6:**
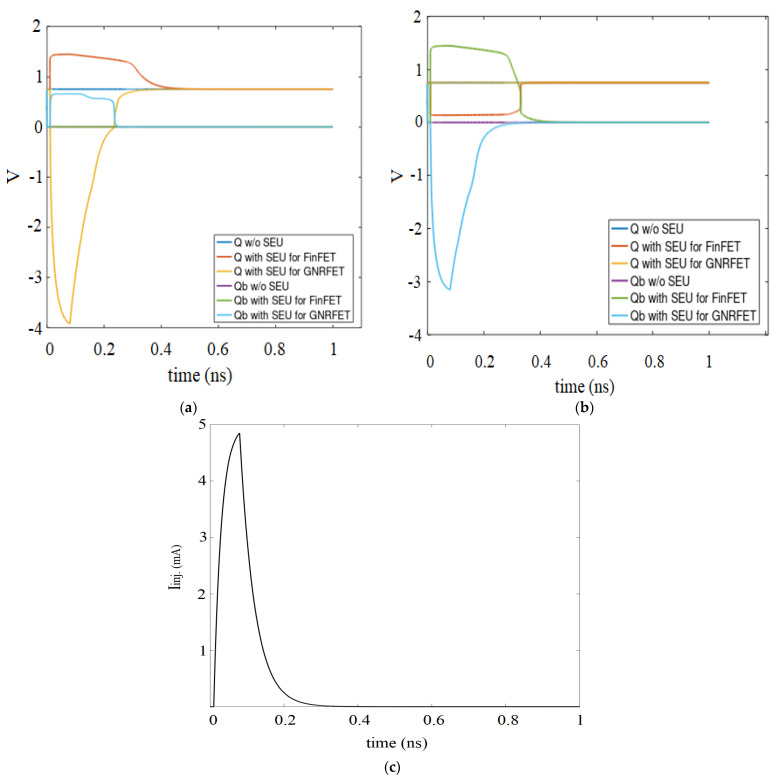
Timing diagram for SRAM with single-event upset (**a**) Node A; (**b**) Node B; (**c**) SEU current pulse.

In Figure 6a, the output Q of an ideal GNRFET recovers faster than FinFET after radiation strike, and both align with Q without SEU after 0.46 ns and 0.51 ns, respectively. Unlike Qb degradation in GNRFET, FinFET Qb is unaffected, and the degradation in Q for GNRFET is also ~2.7X, which implies that it is more sensitive to single-event transient at node A.

On the contrary, both Q and Qb of FinFET SRAM in Figure 6b are largely affected by the radiation effect at node B. The two outputs flip immediately the $Q_{\mathrm{Coll}.}$ due to a charge particle strike exceeds $Q_{\mathrm{Crit}.}$, but it is not enough to make the node lose its recovering potential. There is, however, ~2.16X degradation observed in Qb of GNRFET SRAM. It is expected since technologies that operate with low voltage tend to be less resistant to radiation and have a high soft error rate.

## 4. Stability of Graphene Nano-Ribbon FET SRAM Cell

**Figure 7:**
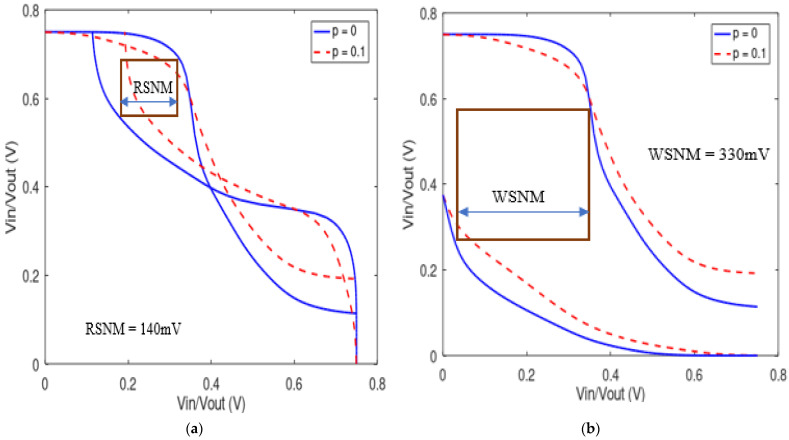
Static noise margin for SRAM (**a**) read; (**b**) write.

Because of aggressive V_DD_ scaling and increased intra-die variability, the two critical metrics of read and write stability of SRAM cells are major concerns. Stability, also known as the static noise margin (SNM), is the minimum voltage noise that can change the state of SRAM at the storage node. Although there are other methods of SNM measurements, the butterfly plot is considered one of the best ways to measure stability. It is a voltage transfer curve of the storage cell or two-inverter circuitries superimposed on each other. We employed the test benches in ref. [12] for measurements, and the results are presented in Figure 7. The read and write SNM are higher than when 10% edge roughness is introduced, i.e., the robustness of SRAM is reduced with LER.

We equally investigated how SNM varies with transistor channel length or technology node and supply voltage V_DD_. An increase in Lch from 10 nm to 25 nm results in a 22.1% and 19.7% increase in read and write stability, respectively. Similarly, the change in V_DD_ from 0.5 V to 0.75 V improves the read SNM by 45 mV and write SNM by 120 mV.

## 5. Conclusions

In this work, transistor-level properties of MOS-GNRFET and PTM nano-MOSFET models are analyzed, and the impact of the level of edge roughness on the performance of GNRFET is also examined. Delay, power, and EDP are evaluated for different values of Lch, Tox, and the probability of edge roughness, p_r_. Subsequently, the response of the single-effect upset on both 6T MOS-GNRFET and FinFET SRAMs is investigated. By using circuit simulation, a transient current is injected at the drains of M2 and M4 successively, and the outputs of SRAM flip due to primary and secondary interactions caused by charged particles. However, both Q and Qb recover from the strike, and the recovery time for GNRFET was estimated and compared with FinFET. More so, it is shown that the SNM of the inverter can be improved by increasing the transistor channel length and/or V_DD_. The static noise margin of SRAM during the read operation is lower than the write SNM, which concludes that SRAM is more vulnerable to flip state or data at the read mode.

## Figures and Tables

**Table 1:** Model and device parameter definitions.

Device Parameter	Description	Value Range
Lch	Channel length	~10–100 nm
Wch	Channel width	0.873–6.36 nm
nRib	Number of GNRs in the device	6–50
Tox	Oxide thickness	0.5–2.5 nm
dop	Doping fraction	0.001–0.0015
p	Edge roughness	0–20%
sp	Spacing between ribbons	1 nm default

**Table 2:** Transistor performance comparison.

Device	pr	I_off_ (A)	I_on_/I_off_	V_DD_ (V)	V_pinch-off_ (mV)
GNRFET	0	1.24 × 10^−8^	1.33 × 10^4^	0.5	404
	0.1	5.21 × 10^−7^	2.59 × 10^1^	0.5	565
CMOS FinFET	-	1.48 × 10^−12^	1.1 × 10^7^	0.75	580

## Data Availability

The data presented in this study are available on request from the corresponding author.
